# Seroepidemiology of Human T-Cell Lymphotropic Virus Type-1 (HTLV1) in Mashhad

**DOI:** 10.5539/gjhs.v6n5p99

**Published:** 2014-05-13

**Authors:** Hamidreza Safabakhsh, Mehrdad Jalalian, Gharib Karimi

**Affiliations:** 1 Blood Transfusion Research Center, High Institute for Research and Education in Transfusion Medicine, Tehran, Iran; 2 Electronic Physician, Mashhad, Iran

**Keywords:** HTLV-I, blood donors, seroprevalence, Mashhad, Iran

## Abstract

**Introduction:**

Human T-cell lymphotropic virus (HTLV-I) is associated with adult T cell leukemia/lymphoma (ATL) and HTLV-I-associated myelopathy/tropical spastic paraparesis (HAM/TSP). The major routes of HTLV-I transmission are mother-to-child, sexual contact, and blood transfusion. Mashhad is one of the main endemic areas in the world for HTLV-I, and minimizing the risk of HTLV-I transmission through blood transfusion is one of the main duties of the Blood Transfusion Center in Mashhad. The aim of this study was to determine the prevalence of HTLV-I in the blood donor population in Mashhad during 2011-2013.

**Methods:**

All the blood donors in Mashhad from March 2011 to April 2013 who were diagnosed with HTLV-I on the ELISA screening test and the Western blot confirmatory test were included in this seroepidemiological study.

**Results:**

From 174,662 blood donors, 327 donors were confirmed to be infected with HTLV-I according to Western blot assay. The seropositive donors ranged in age from 17 to 59, and their mean age was 39.88±10.49 years. The overall prevalence rates of HTLV-I infection were calculated as 0.18% and 0.19%, respectively.

**Conclusion:**

Due to the lower frequency of infection in regular blood donors, younger individuals, and people with higher education levels, the selection of blood donors from these populations should be further considered.

## 1. Introduction

HTLV-I is a member of Retroviridae family and was first isolated in Japan in 1979 from the lymphocyte culture of a young black person with cutaneous T-cell lymphoma. Subsequently, it was reported in other parts of the world. This virus can cause serious diseases, such as adult T-cell leukemia/lymphoma (ATL), HTLV-I-associated myelopathy/tropical spastic paraparesis (HAM/TSP), and uveitis (Bittencourt, 2006). The distribution of infection with this virus is worldwide, but it is endemic in some areas, such as Japan, Taiwan, the Caribbean basin, Central and Southern Africa, parts of the Middle East, and the Southeastern United States (Edlich, 2003). Northeast Iran also is considered to be an endemic area, and the prevalence of infection in the general population of Mashhad was 2.12% (Rafatpanah et al., 2011). In the latest study of blood donors in Mashhad in 2010 and 2011, the prevalence of HTLV-I infection was reported to be 0.26% (Safabakhsh, 2014). In the provinces of West Azerbaijan, Ilam, Hormozgan, and Bushehr, the percentages of blood donors with the virus were 0.34, 0.21, 0.18, and 0.01%, respectively (Abedi, 2009; Falahi, 2008; Khameneh, 2008; Pour Karim, 2004). According to recent studies conducted in other countries, the prevalence of HTLV-I infection in blood donors in Japan, Korea, Turkmenistan, Spain, and Brazil was 1.9, 0.007, 0.27, 0.001, and 0.12%, respectively (Carneiro-Proietti, 2011; Chaudhari, 2009; Korean red cross, 2008; Toro, 2002; Natalia, 1998). The modes of transmission of the virus are mainly through sexual contact with an infected person; mother-to-child, particularly through breastfeeding; and blood transfusion. HTLV-I transmission through blood products containing white blood cells has been reported to range from 44 to 66%, but there have been no reports of transmission through acellular blood products, such as plasma (Hedayati-Moghaddam, 2013). Rarely, HTLV-I transmission has been reported through liver, kidney, and lung transplants (Yara, 2009). Considering the seriousness of the diseases caused by infection with this virus and the fact that Mashhad is one of the most important HTLV-I endemic areas worldwide, laboratory screenings for HTLV-I/II have been performed on donated blood samples in Mashhad and some other areas in Iran since 1994. The prevalence rate of HTLV-I infection in the blood-donor population in Mashhad was reported to be 1.97% in 1995 (Abbaszadegan, 2003; Rezvan, 1996). Since continuous epidemiological studies in endemic areas results in continuous monitoring of the infection trend in the population while updating the information about the infection, continuous study of the prevalence of this virus is necessary. In addition, performing such studies reveals the changes in the prevalence of such infections and the patterns by which the infection spreads over time. This information provides further knowledge and insight that can be used for planning how to manage and control such infections The aim of this study was to investigate the prevalence of HTLV-I infection in blood donors in Mashhad in a two year period from March 2011 to April 2013.

## 2. Materials and Methods

This cross-sectional study was performed using the data from the records of blood donors in the Mashhad Blood Transfusion Center from March 2011 to April 2013 through the census method. All donors who were positive for anti-HTLV-I/II on the ELISA screening test and the Western blot confirmatory test were considered as confirmed positive cases of HTLV-I infection. The demographic data of these individuals (age, gender, marital status, educational level, and the number of blood donations) were obtained, coded, and recorded in the database. The collected data were analyzed using SPSS software (version 17).

## 3. Results

**Figure 1:**
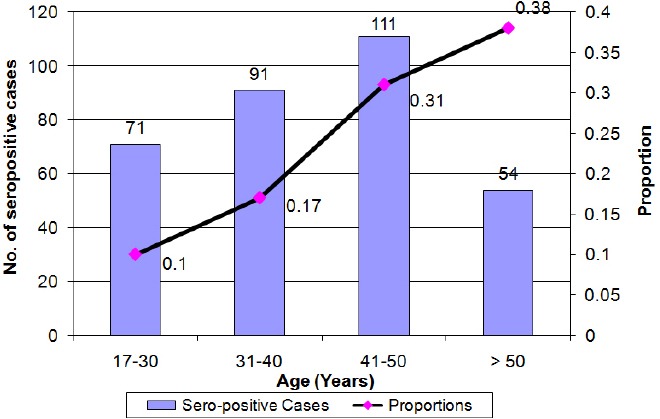
Age distribution of HTLV-I positive cases (bar graph) and prevalence (curve) in blood donors From March 2011 to April 2013

During the study period, 174,662 donors donated blood at the Blood Transfusion Center in Mashhad. Among the donors, 162,094 were male (92.8%) and 12,568 were female (7.2%). Of this number, 327 blood donors were determined to be positive for anti-HTLVI/II in the Western blot confirmatory test, and they formed the seropositive population used for this study. Of the seropositive population, 257 were male (78.6%) and 70 were female (21.4%). In general, the prevalence of the infection among the study’s population was estimated to be 0.18%. The ages of the seropositive donors ranged from 18 to 66, and the mean age was 39.62 ± 10.48 years. The study population was categorized in four age groups, i.e., 17-30, 31-40, 41-50, and above 50 years. Among these four groups, the age group of 41-50 had the largest number of seropositive donors with 111 people (33.9% of the total of seropositive donors). Furthermore, the age-related prevalence of HTLV-I was calculated, and it was observed that the prevalence increased uniformly with the increase in age (Figure 1). The prevalence of infection by gender was calculated to be 0.16% in males and 0.56% in females (Table 1). Of the seropositive donors, 246 individuals had a high school diploma or less (75.2%), and 81 individuals had an academic education (24.8%). In terms of the number of blood donations, 319 of the seropositive individuals were first-time donors (97.6%), seven individuals were repeat donors (2.1%), and one individual was regular donor (0.3%) who was a 41-year-old male with a university degree. The prevalence of the infection over the two years that were studied, including the overall prevalence and the prevalence by gender are presented in Table 1.

**Table 1:** Number of HTLV-I positive cases in Mashhad blood donors

Year		Confirmed positive	Donor population	Prevalence by gender	Total prevalence
1^st^ year	Male	124	80253	0.15%	0.18%
	Female	36	6524	0.55%	
2^nd^ year	Male	133	81841	0.16%	0.19%
	Female	34	6044	0.56%	

Total		327	174662		0.18%

## 4. Discussion

In this study, the overall prevalence of HTLV-I infection in blood donors in Mashhad was 0.18%, which was a decrease compared to the prevalence identified in earlier studies in Mashhad. In the survey that was conducted in 1995, the prevalence of infection in blood donors in Mashhad was 1.97% (Rezvan, 1996). In 2001, another study was published that evaluated 229,037 blood donors over a four-year period in Mashhad, and the prevalence of HTLV-I infection was reported to be 1.16% (Tavanai, 2001). In a 2003 study, 208 of 28,926 blood donors in Mashhad were positive, indicating that the prevalence of HTLV-I was 0.77% (Abbaszadegan, 2003). In another study conducted from 2004 to 2006 on blood donors in Mashhad, the prevalence of infection was estimated to be 0.45% (Tarhini et al., 2009). In a study conducted during the years 2006 to 2008, the prevalence of HTLV-I infection was 0.40% (Hatami, 2012). In the latest study on the population of blood donors in Mashhad, the prevalence of infection was 0.26% (Safabakhsh, 2014). Observing the trend of changes in prevalence of this virus in blood donors in Mashhad over time suggests that the prevalence of this infection has been decreasing since 1995. The most important reasons for this decreasing trend are likely due to 1) the enhancement and improvement of donor selection in the Blood Transfusion Center in Mashhad and 2) the increased awareness among blood donors and the general population of the high-risk behaviors and the routes of transmission of this virus. A series of factors have led to the efficient selection of blood donors, and these factors include holding regular specialized practical and educational courses for technical staff, implementing uniform rules and standards, applying quality and sensitive serological tests, using laboratory kits with higher sensitivity and better quality, increasing the number of regular donations, and the development of better equipment (Rezvan, 2007). Also, the use of different public media, the preparation and publication of information pamphlets, holding public conferences for donors and other people, and holding training conferences for health staff of organizations and agencies has increased awareness of blood donors and other people about infections transmitted by blood.

The comparison of the prevalence of HTLV-I infection in the general population and in the blood donors in Mashhad was enlightening. According to the latest study conducted in Mashhad, 35 of 1654 people tested were positive for HTLV-I infection, suggesting a prevalence of 2.12% in the general population (Rafatpanah, 2011). The prevalence of HTLV-I infection in blood donors in Mashhad (0.18%) was considerably lower than the prevalence of this virus in the general population of the city, which, undoubtedly, is a valid indication of effectiveness of screening policies and proper selection of healthy blood donors. Since donors are selected from the group of healthy people, the low prevalence of HTLV-I infection in blood donors compared to the general population seems reasonable (Hedayati-Moghaddam, 2013).

In the studies conducted on blood donors in other parts of Iran, the prevalence of HTLV-I infection was different from Mashhad. For example, prevalence of the infection in the provinces of Charmahal-Bakhtiari, Western Azerbaijan, Ilam, Hormozgan, Southern Khorasan, and Bushehr was reported as 0.62, 0.34, 0.21, 0.18, 0.04, and 0.01%, respectively (Ghafouri, 2011; Abedi, 2009; Falahi, 2008; Khameneh, 2008; Karimi, 2007; Pour Karim, 2004). The prevalence obtained in our study was less than it is in some parts of the country that are not among the endemic areas, which may be indicative of the effective screening and proper selection of donors in the Blood Transfusion Center in Mashhad. The different prevalences in different studies could be due to differences in the geographic locations, the social and demographic composition, the patterns of behavior of the study population, and, in some studies, inadequate sample size (Chaudhari, 2009).

The prevalence of infection in blood donors in Iran’s neighbor to the northeast, Turkmenistan, which is in the endemic area, is 0.27%, which is consistent with the findings of the present study (Natalia, 1998). In the studies conducted in Iran’s other neighboring countries that are in the non-endemic areas, the prevalence of infection in blood donors in Saudi Arabia, Kuwait, and Turkey was reported to be 0.006%, 0.016%, and 0%, respectively (Sertöz et al., 2010; Ul-Hassan, 2004; Al-Mufti, 1998). This information confirms the geographical cluster of HTLV-I in the areas that have designated as endemic areas (Rezvan, 1996).

Among the countries that have endemic areas, Japan was found to have an overall prevalence of 0.66% in males and 1.02% in females among 1,196,321 blood donors, which are considerably higher than the levels obtained in the present study (Satake, 2012). The prevalence of infection with this virus in blood donors in Brazil was reported to be 0.12%, which is consistent with the results of our study (Carneiro-Proietti, 2011). The prevalence of HTLV-I infection among blood donors in non-endemic countries, such as Greece, Lebanon, and Korea, were reported as 0.013%, 0.028%, and 0.007%, respectively. These values are clearly lower than the levels obtained for donors in the present study (Korean Red Cross, 2008; Tamim, 2008; Zervou et al., 2004). The prevalence of HTLV-I infection among blood donors in non-endemic European countries, such as France (0.004%), Sweden (0.002%), Norway (0.002%), and Spain (0.001%) was significantly lower than the prevalence determined in our study (Proietti, 2005; Toro, 2002; Couroucé, 1993).

In this study, it was observed that the positive rate of anti-HTLV-I increased as age increased, which is consistent with most studies conducted in this field. This observation could be due to the cumulative effects of different contacts over peoples’ lifetimes in the endemic areas (Hedayati-Moghaddam, 2013; Rafatpanah, 2011; Abbaszadegan, 2003). Furthermore, the higher prevalence in females in this study could be due to the higher ability of male to female transmission during sexual contact (Rafatpanah, 2011; Abbaszadegan, 2003). In the present study, the prevalence of HTLV-I infection is higher in married individuals than in unmarried individuals, which is probably due to the higher average age of married people and, therefore, their greater length of exposure to infection with the virus and also transmission through sexual contact (Hedayati-Moghaddam, 2013; Hatami, 2012; Abbaszadegan, 2003). In general, in both endemic and non-endemic areas of HTLV-I infection, higher age, female gender, and lower socioeconomic status are associated with higher infection rate with HTLV-I (Proietti, 2005). The higher prevalence of HTLV-I infection in first-time donors compared to regular donors may be due to the fact that some of the first-time donors donate blood with other motives in mind, such as checking their health or the beneficial effects of blood donation on their health. Also, the prevalence of high risk behaviors in first-time donors could be higher than regular donors. Also, the awareness and education programs relating to the avoidance of high risk behaviors, which are provided to donors by the blood transfusion centers, may result in regular donors having greater awareness about blood safety and avoiding risky behaviors than first-time donors (Jalalian, 2008, 2010; Williams, 1997).

## 5. Conclusions

The findings of this study showed that the prevalence of infection is low in blood donors in Mashhad. The most important factors associated with the decreasing trend in the prevalence of HTLV-I infection among blood donors are improvement of donor selection procedures, continuous training of blood transfusion centers’ personnel, improvement of laboratory screening methods, increased awareness of the general population and donors’ population about the methods of avoiding high risk behaviors, and public awareness regarding the prevention methods of transmission of blood transfusion-transmitted infections (TTI). Considering the lower frequency of infection in regular blood donors, in younger people, and in people with higher educational levels, it is recommended that blood donors in the future be selected from the younger age groups and from people who have higher educational levels.
